# Heart Diseases of Uncertain Etiology: A New Definition of Heart Failure for Epidemiological Studies

**DOI:** 10.3390/jcdd10030132

**Published:** 2023-03-21

**Authors:** Paolo Emilio Puddu, Alessandro Menotti

**Affiliations:** Association for Cardiac Research, Via Voghera, 31, 00182 Rome, Italy

## 1. Introduction

It has been a long time since, in the spectrum of ischemic (IHD) or coronary (CHD) heart diseases, a differentiation was performed between the forms presenting with and those without pain [1]. It was soon recognized that the latter might also be accompanied by certain risk factors, different food consumptions [2,3], and the opposite anatomical picture at autopsy [4,5,6], all elements pointing to potential etiological differences that should be the foundation of differentiating nomenclature and classification. It was also recognized early on [4,5,6] that the size of myocardial scars showed a bimodal distribution: large scars were strongly associated with gross atheroma, the thrombosis of large coronary vessels, and myocardial infarction, while this was not the case for small scars that, among other things, were not more common among cases carrying large scars. The hypothesis was then made that infection, toxic or allergic agents, and other unknown causes could be responsible for cases with multiple small scars that lead to diffuse sclerosis of the myocardium with fibrotic muscle replacement [6] and later to heart failure, which is, however, also the common clinical evolution in terms of the well-known form of congestive heart failure (CHF), of other more classical forms of IHD/CHD in the years following acute and/or repeated infarctions. 

The forms of IHD that present without pain were variously named and eventually classified with the very initial term [2] of Heart Diseases of Uncertain Etiology (HDUE), an operational, arbitrary label given to contrast them with classical, painful forms of IHD/CHD such as myocardial infarction, unstable angina, and sudden death [3,7,8,9,10,11,12,13,14,15,16,17,18,19,20]. Moreover, when clinicians refer to CHF, which was the most common manifestation of what we call HDUE, it is extremely difficult to find etiologic elements that should be contrasted with IHD/CHD based on objective and measured risk factors that in general compete [3,7,8,9,10,11,12,13,14,15,16,17,18,19,20], serving as a further complication of this issue due to nomenclature and classification discrepancies that we will discuss here.

In classifying and defining cardiovascular diseases (CVD) in epidemiological studies, heart diseases are segregated from other major CVD conditions such as stroke and peripheral artery diseases. Although they are subdivided into CHD and other heart diseases, a distinction that is not always clearly stated, CHD is defined by WHO-ICD-9 codes 410–414 [21] or by codes I20–I25 of the WHO-ICD-10 [22], but the meanings of codes called “other forms of ischemic heart disease“, “other acute ischemic heart disease”, and “chronic ischemic heart diseases” are somewhat confusing or at least not explicit, although they represent an improvement of code 412 (chronic ischemic heart disease) of the previous ICD-8 classification [23]. Nowadays, because the WHO ICD-10 classification [22] is universally used in clinical and scientific practice, a reasonable description can be found of the several components of IHD/CHD, but, again, some components of code I25 (chronic ischemic heart disease) are still open to misinterpretations [24,25,26].

When, for epidemiological purposes, in 1979 experts from the International Society and Federation of Cardiology and the World Health Organization met in Rome to provide criteria for the definition and classification of heart diseases [27], it was concluded that the diagnosis of IHD/CHD manifested without the occurrence of typical syndromes (such as angina pectoris, acute ischemic attack, namely the term used by that date, myocardial infarction, sudden death, and in the absence of clear etiology) should be classified outside the typical CHD group. In particular, referring to heart failure, in the absence of clear coronary syndromes, the diagnosis of CHD should remain only presumptive [27]. This concept heavily contradicted a previous position of the investigators of the Evans County population study in the USA, where cases of heart failure in people aged 40 years or more, in the absence of reasonable causes, should be classified as CHD [28]. It is evident from a literature search of risk factors or causes where largely different types of CVD are pooled together, simply because they belong to the same anatomo–physiological system, although likely different from the etiological point of view, that these uncertainties in nomenclature give rise to various consequences. An example of these problems was found in a relatively large investigation on heart failure published in the a journal [29] where, however, the definition of the target element was based just on replying “Yes” by the enrolled individuals to the generic question “Has a doctor or other health professional ever told you that you had congestive heart failure?” (N = 180 of 5598 participants with known status after excluding cancers, pregnancy, and less than 18-year-old people). Definitions given on the basis of these replies might be similar to searching for common etiology and risk factors for the entire digestive system (from the pharynx down to the rectum) simply because the several tracts belong to the same system and the positive question was “Has a doctor or other health professional ever told you that you had digestive troubles?”.

## 2. Observations on HDUE

Our research group, involved in a number of population studies in different countries, recently summarized [20] the results obtained with HDUE in population samples located in three continents from the Seven Countries Study of Cardiovascular Diseases, the Italian Rural Areas (IRA) of the same study, the Gubbio Population study run in a small town of central Italy, and the RIFLE Project run on 45 population samples in various Italian regions in more than 12,000, around 1700, around 3500, and more than 47,000 subjects, respectively.

The first two studies included only men, and the other two covered both genders with variable age ranges but usually middle-aged people. A baseline examination including a variable number and type for personal characteristics and risk factors mainly focused on those of a cardiovascular nature was followed by systematic follow-up for mortality and causes of death and in the IRA also by incidence of major cardiovascular diseases. The duration of follow-up ranged from about 7 years up to 50 years. The main structure of the analyses was made by running multivariable models with cardiovascular events as end points and various types of risk factors as predictors. 

Initially, the criteria for the definition of CHD, mainly for mortality and partly for incidence, were rather flexible and sensitive in order to include many cases, but not so highly specific. Later, the need was felt to use more specific criteria in order to identify true CHD events that eventually included only cases manifested as myocardial infarction, acute ischemic attacks, and sudden coronary death. We disregarded heart diseases that were etiologically defined or very rare, such as congenital and rheumatic heart diseases, non-rheumatic valvular diseases, pericarditis, endocarditis, myocarditis, cardiomyopathies, pulmonary heart diseases, ill-defined heart diseases, and other rare conditions.

The residual heart diseases were characterized by a heterogeneous pool of cases, poorly classified, including hypertensive heart diseases usually not well documented by accompanying left ventricular hypertrophy, cases manifested only as heart failure, arrhythmia, or blocks (defined as symptomatic heart diseases according to the International Classification of Diseases), and cases vaguely classified as chronic or other types of CHD but not accompanied by typical coronary syndromes (and pain) described above. In general, symptomatic heart diseases, chronic CHD, and hypertensive heart disease covered about 65%, 25%, and 10%, respectively, of all HDUE. Incidentally, in preliminary tests, the three components were separately analyzed and proved to have similar characteristics. Initially, this group was called “atypical-or only possible-CHD”, but after a number of comparative analyses versus CHD, this subgroup was called HDUE, a definition used in a very early stage of nomenclature efforts [2].

Detailed results are reported in the original articles [3,7,8,9,10,11,12,13,14,15,16,17,18,19] and the condensed and comparative summary [20], while here we provide the main findings. Several differences were found between the two groups (CHD versus HDUE), with HDUE having a stronger relationship with age, appearing at older ages, having a definitely higher age at death, initially manifesting preferably with non-fatal heart failure, having a flat or inverse relationship with serum cholesterol and HDL cholesterol, and a neutral relationship with vigorous physical activity and healthy diet. The predictive role of blood pressure and cigarette smoking was relatively similar comparing CHD to HDUE. Findings were rather uniform across different locations and countries and different lengths of follow-up. Marginal exceptions were the not significant role of serum cholesterol for CHD in Japan, probably because of the small samples and the limited number of events, and the limited difference in age at death between CHD and HDUE in the RIFLE project likely due to the short follow-up duration, preventing the population to enter definite older ages as needed to calculate the age at death well.

A clear etiology could not be identified for HDUE but few efforts were made in the available literature to identify it, despite its frequency that, in almost extinct male populations of the Seven Countries Study, represents around 10% of total mortality and 20% of CVD mortality [19]. Overall, not only the relationships of serum cholesterol with HDUE and CHD were different but also their baseline mean levels: baseline mean serum cholesterol was 235 mg/dL in future CHD deaths versus 199 mg/dL in future HDUE deaths [10]. This may mean that, everything else being equal, the difference of 36 mg/dL is associated with a delay of about 5 years in the occurrence of a fatal heart disease, CHD coming first, and HDUE only later with a 5 year longer expectancy of life. The main characteristics of HDUE cases are occurrence at older age compared with CHD, no relation with cholesterol levels, and perhaps no association with healthy diet and vigorous physical activity, while some conflicting evidence was shown for cigarette smoking [20].

A major difference [20] between most contributions in the cardiovascular epidemiological field and our own studies is that, for over half of the fatal events in the SCS and IRA, beyond the availability of death certificates, we could also exploit information from repeated field examination (including ECG tracings), clinical records from hospitals and other sources, interviews with family and hospital doctors and with relatives of the subjects, and any other possible sources of information. The same information, apart from death certificates, was available for non-fatal events, not relying on externally determined ICD codes. This approach probably allowed a better identification and classification of CVD and other causes of death, at least within the limits of the epidemiological procedures, anticipating years earlier the concept and structure of the Verbal Autopsy Instruments produced by the WHO many years later [30]. It is critically evident that these methods and ways of defining target events are dramatically more precise than replies given to generic questions such as those quoted above [29].

## 3. HDUE and CHF

Since the majority of our cases of HDUE had CHF as the first manifestation, we initially focused on this condition. We could not find many examples providing the frequency of heart failure of unknown cause, both in clinical and population studies run in Western countries [31,32,33,34,35,36,37,38]. Usually, the majority of cases were attributed to CHD such as in the Evans County experience [28], while those of unknown origins varied from 15 to 37%. Little attention was given to the possible causes but high blood pressure was frequently considered a significant risk factor [36,37,38,39]. This probably means that in Western countries the major cause of heart failure is CHD, while for a substantial proportion of cases a clear etiology cannot be found. The picture seems entirely different in African countries [40,41,42] or other developing countries [43] where CHD is still rare and the major reasons for developing CHF are attributed to hypertension, rheumatic heart disease, and some specific African cardiomyopathies of uncertain etiology.

Instead, in review papers, the possible “causes“ of CHF (in general) were described in a chaotic way, including a mix of established heart diseases, such as myocardial infarction and valvular heart disease, more or less traditional risk factors of heart diseases such as dyslipidemia (classified as a minor risk factor), hypertension, dietary factors, smoking habits, sedentary life styles, diabetes, and obesity, and findings of diagnostic procedures, such as left ventricular hypertrophy, ECG abnormalities, and carotid wall thickness [44,45]. In the abovementioned list, several items have nothing to do with etiology, classic risk factors, or determinants since they are established CVD conditions of different types or partial diagnostic data that, in their natural history, can evolve into CHF.

In other analyses of population studies, including a reappraisal of the Framingham Study [46], and others in the USA [47], Sweden [48], and Japan [49], levels of serum cholesterol were not (or even inversely) correlated with the occurrence of CHF, but only in a Japanese study a clear segregation of ischemic from non-ischemic cases was carried out, showing the presence of an inverse association with CHF only in cases of non-ischemic heart disease and a direct one in cases of CHD (myocardial infarction).

All studies performed in the epidemiologic setting have the inherent limitation of not having taken advantage of techniques such as echocardiography that are nowadays essential to differentiate clinical forms of CHF, such as those with reduced versus preserved ejection fractions [50], and clearly these elements may not be used to help correlate with HDUE as defined here. Interestingly enough, in a classical epidemiologic investigation, yet with the definition limitations outlined above, it was reported in a journal [29] that the atherogenic index of plasma (AIP), calculated using the formula of log (triglyceride/high-density lipoprotein cholesterol), accurately represents not only the relationship between protective and atherogenic lipoproteins, but also potentially serves as a strong predictor of atherosclerosis and CHD and it is negatively correlated with CHF prevalence. In the fully adjusted model, after controlling for confounders, each unit of increased AIP was associated with a 72% decreased risk of CHF (aHR = 0.28; 95% CI: 0.10–0.78; *p* = 0.0154). Moreover, in the highest quartile of AIP age, male sex, total and LDL cholesterol, hs-CRP, triglycerides, and uric acid were significantly the highest whereas HDL cholesterol and high education were the lowest, which is globally in line with what we have observed for HDUE [20].

## 4. Conclusions

Although autoptic differences were seen early and clearly in painful versus painless IHD/CHD forms [4,5,6], the latter closer to HDUE [3,7,8,9,10,11,12,13,14,15,16,17,18,19,20], these were not stressed later on if we consider the large terminology uncertainties that remained in the various editions of the international nomenclature manuals and reports [21,22,23,24,25,26,27,28] and the poor attention given in the literature to these differences [29,31,32,33,34,35,36,37,38,39,40,41,42,43,44,45,46,47,48,49].

HDUE included cases classified as symptomatic heart diseases, ill-defined hypertensive heart diseases, and cases of “chronic coronary heart diseases” in the absence of typical coronary syndromes. The pool of these cases is quite large and in terms of mortality covers around 20% of all CVD mortality and around 10% of all-cause mortality in population settings followed-up for 50 years. This group of heart diseases, when compared with typical CHD, is characterized by a stronger association with age, (i.e., appearing at older ages and having a greater age at death), the absence of or an inverse or flat association with serum cholesterol, and no relationship with dietary habits and physical activity. Blood pressure and smoking habits (the latter with some uncertainties) were directly associated with HDUE. In terms of possible etiology or at least of relevant risk factors, the most evident fact is the absence of serum cholesterol as a risk factor for HDUE, tending to exclude a gross coronary atherosclerosis at the level of conductive vessels. This fact might suggest the existence of risk competition that in the IRA study was clearly documented [18]: using a predictive model specific for the evaluation of competing risks (Fine-Gray variant of the Cox model) showed the critical role of serum cholesterol that was directly associated with CHD events and inversely associated with other causes of death in a 50-year follow-up. We have in preparation a competing risk investigation in the context of the SCS at large that might be the first example of this type to index a documented risk assessment in HDUE versus IHD/CHD forms. The nomenclature term HDUE (and its etiologic difference) might thus be ascertained on a more solid foundation were the initial differences [18] versus painful forms to be confirmed.

The etiology of HDUE, although probably multiple, is still vague and elusive, and the need of a systematic search seems of great importance. Future investigations need to be stimulated, as we believe it essential to segregate HDUE from CHD when relationships are investigated among risk factors [51] and outcomes either individually or when multiple definitions (such as in the case of CVD) are adopted for both mortality and incidence [52]. In fact, mixing up HDUE and IHD/CHD cases might impact, by dilution, results and conclusions and impinge upon the relevance that older and new risk factors might have and/or on their capacity to predict outcomes.

## References

[1] Puddu V., Menotti A. (1965). Unsicherheit der Grenzen und diagnostischen Kriterien der “ischämischen Kardiopathie”. Arztl. Forsch..

[2] Menotti A., Moschini-Antinori E., Splendiani G. (1963). Heart diseases in Tripolitania. A clinical and statistical study. Mal. Cardiovasc..

[3] Keys A., Blackburn H., Menotti A., Buzina R., Mohacek I., Karvonen M.J., Punsar S., Aravanis C., Corcondilas A., Dontas A.S. (1970). Coronary heart disease in seven countries. Circulation.

[4] Schwartz C.J., Mitchell J.R. (1962). The relation between myocardial lesions and coronary artery disease. I. An unselected necropsy study. Br. Heart J..

[5] Mitchell J.R., Schwartz C.J. (1963). The relation between myocardial lesions and coronary artery disease ii. A selected group of patients with massive cardiac necrosis or scarring. Br. Heart J..

[6] Anonymous (1963). Myocardial Fibrosis. Br. Med. J..

[7] Aravanis C., Blackburn H., Buzina R., Djordjevic B.S., Dontas A.S., Fidanza F., Karvonen M.J., Kimura N., Menotti A., Mohacek I., Keys A. (1980). Seven Countries Study. A Multivariate Analysis of Death and Coronary Heart Disease.

[8] Kromhout D., Menotti A., Blackburn H. (2002). Prevention of coronary heart disease. Diet, Lifestyle and Risk Factors in the Seven Countries Study.

[9] Menotti A., Blackburn H., Seccareccia F., Kromhout D., Nissinen A., Karvonen M., Fidanza F., Giampaoli S., Buzina R., Mohacek I. (1998). Relationship of some risk factors with typical and atypical manifestations of coronary heart disease. Cardiology.

[10] Menotti A., Lanti M., Maiani G., Kromhout D. (2005). Forty-year mortality from cardiovascular diseases and their risk factors in men of the Italian rural areas of the Seven Countries Study. Acta Cardiol..

[11] Menotti A., Lanti M., Nedeljkovic S., Nissinen A., Kafatos A., Kromhout D. (2005). The relationship of age, blood pressure, serum cholesterol and smoking habits with the risk of typical and atypical coronary heart disease death in the European cohorts of the Seven Countries Study. Int. J. Cardiol..

[12] Menotti A., Puddu P.E., Lanti M., Kromhout D., Tolonen H., Parapid B., Kircanski B., Kafatos A., Adachi H. (2013). Epidemiology of typical coronary heart disease versus heart disease of uncertain etiology (atypical) fatalities and their relationships with classic coronary risk factors. Int. J. Cardiol..

[13] Puddu P.E., Terradura Vagnarelli O., Mancini M., Zanchetti A., Menotti A. (2014). Typical and atypical coronary heart disease deaths and their different relationships with risk factors. The Gubbio residential cohort Study. Int. J. Cardiol..

[14] Menotti A., Puddu P.E. (2015). Lifetime prediction of coronary heart disease and heart disease of uncertain etiology in a 50-year follow-up population study. Int. J. Cardiol..

[15] Puddu P.E., Menotti A. (2015). Natural history of coronary heart disease and heart disease of uncertain etiology: Findings from a 50-year population study. Int. J. Cardiol..

[16] Menotti A., Puddu P.E., Maiani G., Catasta G. (2015). Lifestyle behaviour and lifetime incidence of heart diseases. Int. J. Cardiol..

[17] Menotti A., Puddu P.E., Maiani G., Catasta G. (2016). Cardiovascular and other causes of death as a function of lifestyle in a quasi-extinct middle-aged male population. A 50-year follow-up study. Int. J. Cardiol..

[18] Puddu P.E., Piras P., Menotti A. (2017). Lifetime competing risks between coronary heart disease mortality and other causes of death during 50 years of follow-up. Int. J. Cardiol..

[19] Menotti A., Puddu P.E., Tolonen H., Adachi H., Kafatos A., Kromhout D. (2019). Age at death of major cardiovascular diseases in 13 cohorts. The seven countries study of cardiovascular diseases 45-year follow-up. Acta Cardiol..

[20] Menotti A., Puddu P.E. (2019). Epidemiology of heart disease of uncertain etiology: A population study and review of the problem. Medicina.

[21] WHO International Classification of Diseases (WHO-ICD-9) World Health Organization (1975). International Classification of Diseases and Causes of Death.

[22] WHO International Classification of Diseases (WHO-ICD-10) (1992). International Statistical Classification of Diseases and Related Health Problems.

[23] WHO International Classification of Diseases (WHO-ICD-8) World Health Organization (1965). International Classification of Diseases and Causes of Death.

[24] World Health Organization (1958). Classification des Lésions D’athérosclerose.

[25] World Health Organization (1959). Hypertension and Coronary Heart Disease. Classification and Criteria for Epidemiological Studies.

[26] Burgess A.M., Feifar Z., Kagan A. (1963). Hypertension arterielle et cardiopathie ischémiques. Comparibilité des études Epidemiologiques.

[27] Nomenclature and Criteria for Diagnosis of Ischemic Heart Disease (1979). Report of the Joint International Society of Cardiology and Federation of Cardiology/World Health Organization task force on standardization of clinical nomenclature. Circulation.

[28] Bartel A., Heyden S., Tyroler H.A., Tabesh E., Cassel J.C., Hames C.G. (1971). Electrocardiographic predictors of coronary heart disease. Arch. Intern. Med..

[29] Xue J., He L., Xie H., Xie X., Wang H. (2022). An inverse correlation between the atherogenic index of plasma and heart failure: An analysis of the National Health and Nutrition Examination Survey 2017–March 2020 pre-pandemic data. J. Cardiovasc. Dev. Dis..

[30] WEB Site (2015). Verbal Autopsy Standards. The 2012 WHO Verbal Autopsy Instruments.

[31] Murdoch D.R., Love M.P., Robb S.D., McDonagh T.A., Davie A.P., Ford I., Capewell S., Morrison C.E., McMurray J.J. (1998). Importance of heart failure as a cause of death. Changing contribution to overall mortality and coronary heart disease mortality in Scotland 1979–1992. Eur. Heart J..

[32] Mendy V.L., Vargas R., Payton M. (2017). Trends in mortality rates by subtypes of heart disease in Mississippi, 1980–2013. BMC Cardiovasc. Disord..

[33] Fabbri G., Gorini M., Maggioni A.P., Cacciatore G., Di Lenarda A. (2006). Italian Network on Congestive Heart Failure: Ten-year experience. G. Ital. Cardiol..

[34] Parameshwar J., Shackell M.M., Richardson A., Poole-Wilson P.A., Sutton G.C. (1992). Prevalence of heart failure in three general practices in north west London. Br. J. Gen. Pract..

[35] Mair F.S., Crowley T.S., Bundred P.E. (1996). Prevalence, aetiology and management of heart failure in general practice. Br. J. Gen. Pract..

[36] Dawber T.R. (1980). The Framingham Study. The Epidemiology of Atherosclerotic Disease.

[37] Kannel W.B. (2000). Vital epidemiologic clues in heart failure. J. Clin. Epidemiol..

[38] Remes J., Reunanen A., Aromaa A., Pyörälä K. (1992). Incidence of heart failure in eastern Finland: A population-based surveillance study. Eur. Heart J..

[39] Mahmood S.S., Levy D., Vasan R.S., Wang T.J. (2014). The Framingham Heart Study and the epidemiology of cardiovascular diseases: A historical perspective. Lancet.

[40] Mayosi B.M. (2007). Contemporary trends in the epidemiology and management of cardiomyopathy and pericarditis in sub-Saharan Africa. Heart.

[41] Falase A.O., Ogah O.S. (2012). Cardiomyopathies and myocardial disorders in Africa: Present status and the way forward. Cardiovasc. J. Afr..

[42] Sliwa K., Mayosi B.M. (2013). Recent advances in the epidemiology, pathogenesis and prognosis of acute heart failure and cardiomyopathy in Africa. Heart.

[43] Kwan G.F., Jean-Baptiste W., Cleophat P., Leandre F., Louine M., Luma M., Benjamin E.J., Mukherjee J.S., Bukhman G., Hirschhorn L.R. (2016). Descriptive epidemiology and short-term outcomes of heart failure hospitalisation in rural Haiti. Heart.

[44] Bui A.L., Horwich T.B., Fonarow G.C. (2011). Epidemiology and risk profile of heart failure. Nat. Rev. Cardiol..

[45] Khatibzadeh S., Farzadfar F., Oliver J., Ezzati M., Moran A. (2013). Worldwide risk factors for heart failure: A systematic review and pooled analysis. Int. J. Cardiol..

[46] Ho K.K.L., Pinsky J.L., Kannel W.B., Levy D. (1993). The epidemiology of heart failure: The Framingham Study. J. Am. Coll. Cardiol..

[47] Gottdiener J.S., Arnold A.M., Aurigemma G.P., Polak J.F., Tracy R.P., Kitzman D.W., Gardin J.M., Rutledge J.E., Boineau R. (2000). Predictors of congestive heart failure in the elderly: The Cardiovascular Health Study. J. Am. Coll. Cardiol..

[48] Wilhelmsen L., Rosengren A., Eriksson H., Lappas G. (2001). Heart failure in the general population of men. Morbidity, risk factors and prognosis. J. Intern. Med..

[49] Nago N., Ishikawa S., Goto T., Kayaba K. (2011). Low cholesterol is associated with mortality from stroke, heart disease, and cancer: The Jichi Medical School Cohort Study. J. Epidemiol..

[50] Gavina C., Seabra Carvalho D., Valente F., Bernardo F., Jorge Dinis-Oliveira R., Santos-Araújo C., Taveira-Gomes T. (2022). 20 years of real-world data to estimate the prevalence of heart failure and its subtypes in an unselected population of integrated care units. J. Cardiovasc. Dev. Dis..

[51] Scicchitano P., Iacoviello M., Passantino A., Gesualdo M., Trotta F., Basile M., De Palo M., Guida P., Paolillo C., Riccioni G. (2022). Plasma levels of intact parathyroid hormone and congestion burden in heart failure: Clinical correlations and prognostic role. J. Cardiovasc. Dev. Dis..

[52] Kerexeta J., Larburu N., Escolar V., Lozano-Bahamonde A., Macía I., Beristain Iraola A., Graña M. (2023). Prediction and analysis of heart failure decompensation events based on telemonitored data and artificial intelligence methods. J. Cardiovasc. Dev. Dis..

